# Gastric Cancer Risk Was Associated with Dietary Factors Irritating the Stomach Wall: A Case–Control Study in Korea

**DOI:** 10.3390/nu14112233

**Published:** 2022-05-27

**Authors:** Sang Young Kim, Jung Hyun Kwak, Chang Soo Eun, Dong Soo Han, Yong Sung Kim, Kyu Sang Song, Bo Youl Choi, Hyun Ja Kim

**Affiliations:** 1 Department of Food and Nutrition, Gangneung-Wonju National University College of Life Science, 7 Jukheon-gil, Gangneung-si 25457, Korea; sangaustin@gmail.com (S.Y.K.); hyun4615@hanmail.net (J.H.K.); 2 Division of Gastroenterology, Department of Internal Medicine, Hanyang University Guri Hospital, 153 Gyeongchun-ro, Guri-si 11923, Korea; cseun@hanyang.ac.kr (C.S.E.); hands@hanyang.ac.kr (D.S.H.); 3 Functional Genomics Institute, PDXen Biosystems Co., ETRI Convergence Commercialization Center, 218 Gajeong-ro, Yuseong-gu, Daejeon 34129, Korea; yskakim@gmail.com; 4 Department of Pathology, Chungnam National University College of Medicine, 99 Daehak-ro, Yuseong-gu, Daejeon 34134, Korea; qsong@cnu.ac.kr; 5 Department of Preventive Medicine, Hanyang University College of Medicine, 222 Wangsimni-ro, Seongdong-gu, Seoul 04763, Korea; bychoi@hanyang.ac.kr

**Keywords:** case–control study, gastric cancer, meal frequency, overeating, preference

## Abstract

The incidence of gastric cancer is high in Korea, and dietary factors are important risk factors for gastric cancer. This study examined whether gastric cancer risk was related to dietary factors that directly irritate the stomach wall. This case–control study consisted of 308 matched pairs of gastric cancer cases and controls recruited from 2002 to 2006 at two hospitals in Korea. Dietary assessments were completed using a food frequency questionnaire and a dietary habit questionnaire. Gastric cancer risk was increased for high meal frequency of >3 vs. low meal frequency of ≤3 times per day, overeating vs. not overeating, and preferred vs. not preferred spicy or salty foods. Furthermore, participants with dietary factors of high meal frequency, overeating, and preference for spicy or salty foods elevated the risk of gastric cancer compared to those with low meal frequency, not overeating, and not preferring spicy or salty foods, simultaneously. In conclusion, gastric cancer risk was significantly increased in people with dietary factors that irritate the stomach wall, such as high meal frequency, overeating, and preference for spicy or salty foods.

## 1. Introduction

The incidence of gastric cancer ranked fifth, with 1,089,103 newly diagnosed cases based on the global cancer statistics 2020 [1]. The incidence of gastric cancer in Korea was the highest in the world [2], at a rate of 57.4/100,000 persons in 2019 [3].

Various risk factors for gastric cancer have been identified, including diet, lifestyle of alcohol drinking and smoking, genetic talent, family history, and *Helicobacter pylori (H. pylori*) infection [4]. Among the risk factors for gastric cancer, it has been reported that the dietary factor of consumption of fruits, vegetables, and vitamin C decreased gastric cancer risk, but habitual salt intake increased the risk [5]. In addition to these dietary factors, there might be other dietary factors that can directly affect gastric disease by irritating the gastric wall. High meal frequency can stimulate the continued secretion of gastric acid for digestion, and a high state of gastric acid can elevate the risk of several gastric diseases, including peptic ulcer disease [6]. Moreover, the main staple food is rice, and Koreans sometimes consume cereal, bread, noodles, and dumplings as staple food [7]. A high frequency of meals mirrors a high intake of these staple foods. Previous studies have demonstrated an increased risk of gastric cancer for the high consumption of staple foods, including refined grains [8] and noodles and dumplings [9], and frequent consumption of noodles and bread [10]. For the refined grain intake, there was a significant increase in the relationship with gastric cancer risk (OR = 1.36, 95% CI = 1.21–1.54), specifically, a large quantity of 1.63 (95% CI = 1.49–1.79) and a moderate quantity of 1.28 (95% CI = 1.18–1.39) [8]. Additionally, there was a significant association between gastric cancer risk and a high intake of noodles and dumplings (OR = 1.65, 95% CI = 1.03–2.63) [9]. For the frequent intake of noodles and bread, the highest quartile was related to the increased risk of gastric cancer in men (OR = 1.50, 95% CI = 1.10–2.00) [10]. In addition, overeating can be both detrimental to the stomach and increase the risk of gastric cancer. This may be due to physical damage to the gastric mucosa caused by repetitions of compulsory expansion of the stomach lining [11]. Furthermore, Koreans traditionally prefer salty and spicy foods. It is widely known that salty diets and spicy food intake have been included risk factors for gastric cancer [4]. One plausible reason is that continued consumption of salty and spicy foods may irritate the stomach wall and contribute to the development of gastric cancer [12].

Thus, this case–control study aimed to investigate whether gastric cancer risk is associated with dietary factors that directly irritate the stomach wall, including meal frequency, overeating, preference for salty or spicy foods, and combinations of these factors among the Korean population.

## 2. Materials and Methods

### 2.1. Study Population

Participants were selected from patients who visited Chungnam National University Hospital and Hanyang University Guri Hospital between December 2002 and September 2006. The inclusion criteria were as follows: (1) participants aged 20–79 years, (2) cases confirmed by histopathological examination, and (3) controls who did not have gastric cancer through gastroscopy in the same hospital during the same period. A total of 440 cases and 485 controls were recruited. We excluded participants with a daily energy intake of <500 kcal (5 cases and 1 control) or >5000 kcal (3 cases and 7 controls). Cases and controls were matched at a 1:1 ratio for sex, visiting hospital, age within 5 years, and study admission period within 1 year. In this process, 124 cases and 169 controls were excluded because there were no matched participants according to the 1:1 matching criterion. Finally, 308 pairs (124 pairs from Chungnam National University Hospital and 184 pairs from Hanyang University Guri Hospital) were included in this study. All participants provided written informed consent, and the study protocol was approved by the institutional review board of Hanyang University Medical Center (IRB no. 2003-4).

### 2.2. Data Collection

A detailed description of the data collection in this study has been previously published [9]. Due to the slight change in questionnaire items during the study period, the participants were classified into the 1st (December 2002 to August 2003) and 2nd (October 2003 and September 2006) stages. The questionnaire consisted of sociodemographic characteristics, anthropometric factors, behavioral factors, disease status, and family history. The tumor-node-metastasis (TNM) stages for gastric cancer were divided into four groups as stage I, II, III, and IV according to the TNM staging system of the American Joint Committee on Cancer (AJCC) [13]. The presence of *H. pylori* infection was tested using the Campylobacter-like organism (CLO) test kit (Product No: 60480; Kimberly-Clark/Ballard Medical Products, Draper, UT, USA), a rapid urea degradation test with high sensitivity and specificity [14]. This test was performed only when tissue samples were collected.

### 2.3. Dietary Data

Data on dietary factors were collected using a quantitative food frequency questionnaire (FFQ), a slightly revised version of the validated FFQ. The FFQ in the present study was specifically described in our previous study [15]. All questionnaires were administered by well-trained interviewers, and the frequency and amount of food consumed during the 12 months from 3 years earlier were determined to assess remote dietary intake. The reason for conducting the remote dietary intake was that the patients may have changed their diet because of gastric symptoms, even before gastric cancer diagnosis. To assist in recalling the remote dietary intake, the interviewers asked the participants about their diet changes before 3 years compared at the time of the interview. The FFQs used for the 1st and 2nd stages differed slightly regarding survey items and frequency. Specifically, the FFQ for the 1st stage was composed of open questions for frequency per month, week, or day for 102 food or dish items. The 2nd FFQ included intake frequency as nine categories (never or less than once a month; 1–3 times a month; 1 time a week; 2–4 times a week; 5–6 times a week; once a day; 2–3 times a day; 4–5 times a day; and ≥6 times a day) for 115 foods or dish items. Just for the convenience of recall, there were more food items in the second stage, due to the subdivision of several food items in the first stage. Serving sizes of each food item were answered as open-ended for both the FFQs.

Meal frequency was assessed by substituting the frequency of staple foods. We extracted the same food items for the first stage based on the second stage, and staple foods consisted of rice and noodles and dumplings: (1) rice was composed of seven foods including white rice, multigrain rice, rice with beans, fried rice, bibimbap (mixed rice with various vegetables, meat, and chili pepper paste), gimbap (rice and ingredients such as vegetables, egg, ham that are rolled in a dried sheet of seaweed), and rice porridge; (2) noodles and dumplings was composed of eight dishes including guksu (noodles with broth), bibim-guksu (mixed cold noodles without broth), ramyeon (instant noodles), jajangmyeon (Chinese noodles with black bean sauce), jjamppong (Chinese noodles with vegetables, seafoods, and spicy broth), sujebi and kal-guksu (hand-pulled dough soup and handmade noodle soup), dumplings and dumpling soups, and tteokguk (sliced rice cake soup).

In addition, a dietary habit questionnaire was conducted through an interview, and three dietary questions were selected: (1) “the amount of rice generally served at restaurants is insufficient” to evaluate whether participants overeat or not (overeating); (2) “I like spicy foods” and (3) “I like salty foods” to assess food that irritates the stomach wall (preference for spicy or salty foods). There were five answer choices: definitely, probably, possibly, probably not, and definitely not. The answer choices of possibly, probably not, and definitely not were classified as not overeating, not preferred spicy foods, and not preferred salty foods, respectively. The definitely and probably answer choices were grouped as overeating, preferred spicy foods, and preferred salty foods, respectively. Preference for spicy foods and preference for salty foods were combined as one dietary factor. Specifically, a preferred group was classified when the participants preferred spicy foods, salty foods, or both. Furthermore, each combination of dietary factors was also constructed.

Additionally, a score of 0 was assigned to low meal frequency (≤3 times/day), not overeating, and not preferred spicy or salty foods, respectively. A score of 1 indicated a high meal frequency (>3 times/day), overeating, and preference for spicy or salty foods, respectively. Finally, each score from the three categories was summed. The total score ranged from 0 to 3 as whole numbers, with a lower score presented as healthier and a higher score as unhealthier.

### 2.4. Statistical Analysis

Statistical analyses were performed using SAS (9.4 version; SAS Institute Inc., Cary, NC, USA). Categorical and continuous variables are presented as frequencies (percentages) and mean values ± standard deviation (SD), respectively. To compare the general characteristics of the cases and controls, the chi-square test was used for categorical variables, and the *t*-test was used to analyze continuous variables. The odds ratios (ORs) and 95% confidence intervals (95% CIs) for the risk of gastric cancer were calculated using multivariable logistic regression, specifically, a code of 1 for the dependent variable as gastric cancer cases and a code of 0 as controls. The model was adjusted for known confounding factors, as sex (men or women), age (years, continuous), body mass index (≤22.9, 23.0–24.9, ≥25.0 kg/m^2^, or missing), family history (first-degree relatives) of gastric cancer (no or yes), hospital (Chungnam National University Hospital or Hanyang University Guri Hospital), education (≤elementary school, middle school, high school, ≥college, or missing), smoking (never, past, or current smoker), drinking (never, past, <20 g/day for women or <40 g/day for men, or ≥20 g/day for women or ≥40 g/day for men), *H. pylori* infection (negative, positive, or missing), fruit intake (g/day, continuous), and vegetable intake (g/day, continuous).

## 3. Results

### 3.1. General Characteristics

Table 1 shows the general characteristics of the gastric cancer cases and controls. A total of 308 pairs of cases and controls were included, including 414 men (67.2%) and 202 women (32.8%). The average ages of the cases and controls were 56.9 ± 11.9 years and 56.2 ± 11.9 years, respectively. The cases consisted of a lower proportion of obese (22.1%) and a higher proportion of family history of gastric cancer (15.3%) compared to the controls (32.5% and 9.7%, respectively). The majority of the cases were TNM stage I (46.4%) (data not shown). There were no significant differences between the cases and controls regarding education and smoking status. However, the cases involved more in the drinking status ≥20 g/day for women or ≥40 g/day for men (22.7%) compared to the controls (18.5%) (*p* = 0.036). Additionally, the percentage of controls with *H. pylori* infection was higher (45.8%) than the cases (31.5%) (*p* < 0.001). There were more consumptions of total energy for the cases (1869.6 ± 740.5 kcal/day) than the controls (1698.9 ± 619.6 kcal/day) (*p* = 0.002).

### 3.2. Gastric Cancer Risk by General Risk Factors

Table 2 shows the risk of gastric cancer regarding general risk factors after adjusting for confounding factors. Gastric cancer risk was decreased for obese versus underweight and normal participants (OR = 0.52, 95% CI = 0.35–0.78), but increased for participants with a family history of gastric cancer (OR = 1.74, 95% CI = 1.04–2.91). After adjusting for confounding factors, smoking and alcohol drinking were not significantly associated with gastric cancer risk. However, a decreased risk of gastric cancer was observed in participants with *H. pylori* infection than those without *H. pylori* infection (OR = 0.42, 95% CI = 0.27–0.64).

### 3.3. Gastric Cancer Risk by Dietary Factors Irritating the Stomach Wall

Table 3 presents the gastric cancer risk according to dietary factors that irritate the stomach wall. A high meal frequency of >3 times/day showed significantly increased gastric cancer risk than a low meal frequency of ≤3 times/day (OR = 1.96, 95% CI = 1.38–2.79). In addition, gastric cancer risk was elevated for overeating (high amount of rice intake) vs. not overeating (low amount of rice intake) (OR = 1.96, 95% CI = 1.25–3.07). Additionally, the risk of gastric cancer was increased for the preferred group of spicy or salty foods (OR = 1.54, 95% CI = 1.06–2.22) compared to the non-preferred group.

### 3.4. Gastric Cancer Risk by Combinations of Dietary Factors Irritating the Stomach Wall

Table 4 presents the risk of gastric cancer by combinations of dietary factors that irritate the stomach wall. An increased risk of gastric cancer was identified for high meal frequency/high amount of rice intake (OR = 5.40, 95% CI = 2.71–10.75) and high meal frequency/low amount of rice intake (OR = 1.74, 95% CI = 1.17–2.58), compared to low meal frequency/low amount of rice intake. Additionally, elevated gastric cancer risks were observed for high meal frequency/preference for spicy or salty foods (OR = 2.73, 95% CI = 1.62–4.61) compared to low meal frequency/not preferred spicy or salty foods. For the combinations of overeating with the preference for spicy or salty foods, gastric cancer risk significantly increased for the high amount of rice intake/preferred group (OR = 2.81, 95% CI = 1.56–5.05) and the low amount of rice intake/preferred group (OR = 1.53, 95% CI = 1.02–2.29).

### 3.5. Gastric Cancer Risk by Total Scores of Dietary Factors Irritating the Stomach Wall

Table 5 describes the gastric cancer risk according to the total scores of dietary factors that irritate the stomach wall. Compared to a score of 0 as a healthier score, the risk of gastric cancer was increased for scores of 3 (OR = 6.35, 95% CI = 2.73–14.79) and 2 (OR = 2.39, 95% CI = 1.38–4.14).

## 4. Discussion

This study found that the risk of gastric cancer was significantly increased in participants who frequently had meals, overate, and preferred spicy or salty foods, respectively, as well as their combinations. Moreover, participants with all these dietary factors had an elevated gastric cancer risk.

In Korea, people consume various staple foods as meals, including rice, noodles, and dumplings [16]. However, frequent meals cause the stomach to work continuously because food remains in the stomach longer. This can lead to increased reflux of mixed liquid–gas and acid reflux exposure durations [17]. In addition, the Korean diet mainly consists of carbohydrates, and a high meal frequency can result in high carbohydrate intake. A previous study demonstrated that a high-carbohydrate diet could cause acid reflux [17] and increase cell proliferation and tumor growth caused by elevating aerobic glycolysis or activating the insulin-like growth factor-1 (IGF-1) axis [18]. These adverse health outcomes associated with a high frequency of meals possibly elevate the risk of gastric cancer. In the present study, high meal frequency, which exceeds the recommendation of three meals per day in Korea [19], increased the risk of gastric cancer.

Furthermore, gastric cancer risk was increased for participants who overate in the present study. Overeating forces the stomach lining to expand, which can cause physical damage to the gastric mucosa [11]. Additionally, a significant amount of food eaten at once requires more enzymes and hormones to digest foods, such as increasing hydrochloric acid. Excessive production of hydrochloric acid in the stomach can contribute to a cause of peptic ulcer disease, gastroesophageal reflux disease, and gastrointestinal bleeding [6,20]. Therefore, repeated overeating can significantly increase the risk of gastric cancer.

In addition, people who prefer spicy or salty foods may be more exposed to an increased risk of stomach cancer. First, it is plausible that capsaicin contained in spicy foods acts as a carcinogenic agent, causing mucosal damage [21]. Moreover, spicy foods can worsen symptoms of gastritis or stomach ulcers that induce heartburn, stomach pain, and diarrhea [22]. A meta-analysis showed that the risk of gastric cancer was increased with high spicy food consumption (OR = 2.16, 95% CI = 1.26–3.71) [23]. Second, high sodium intake can cause gastritis [24], and high intra-gastric sodium concentration can induce mucosal inflammation and damage by destroying the mucosal barrier [25]. Previous studies reported an elevated gastric cancer risk in the high sodium consumption group (relative risk = 1.68, 95% CI = 1.17–2.41) [26] as well as the salty preference group (hazard ratio = 1.10, 95% CI = 1.04–1.16) [27]. The present study also found an elevated risk of gastric cancer in participants who preferred spicy or salty foods.

Moreover, considering the adverse effects of each dietary factor itself on the stomach wall, it is plausible that their combination increases the risk of gastric cancer. Among these combinations, it is notable that participants who had meals frequently and overate showed the highest risk of gastric cancer. Frequent overeating causes food to stay in the stomach for a long time by repeating the process of overfilling the stomach with food, which can be converted to fat and contribute to overweight status or obesity. This condition can increase insulin resistance and hyperinsulinemia, which elevates IGF-1 availability [28]. A meta-analysis found that overweight and obesity were linked to elevating gastric cancer risk [29]. Frequent meals and overeating can, respectively, cause adverse effects on the stomach; however, frequent overconsumption of foods can critically elevate the risk of gastric cancer.

Lastly, when the scores of each dietary factor were summed, higher scores (unhealthier scores) were linked to an increased risk of gastric cancer. In more detail, the maximum score of 3, which means that these participants were more likely to have a high meal frequency, overeat, and prefer spicy or salty foods, simultaneously, showed the highest risk of gastric cancer (OR = 6.35, 95% CI = 2.73–14.79) in the present study. Due to the simultaneous effect of these dietary factors irritating the stomach, the gastric mucosal barrier (important in protecting the stomach) can be broken, which diffuses pepsin and acid in the opposite direction into the tissues and secretes more pepsin and acid [30]. Additionally, this can lead to diminishing flow of mucosal blood and gastric motility, furthermore, causing gastric ulcers by damaging submucosal capillaries and connective tissue from the acid [30]. Thus, people, who have all three dietary factors maintaining it continuously for a long term, can exacerbate the adverse symptoms to the stomach and ultimately develop gastric cancer.

This study has several strengths. Each participant was interviewed without disclosing the disease status after endoscopy. The controls were also recruited from the same hospital within the same period, and the absence of gastric cancer was confirmed through gastroscopy. However, multiple limitations exist in this study: (1) the sample was not representative of the Korean population, as recruitment of the participants occurred only at two hospitals in Korea; (2) the questionnaires for the first and second FFQs were slightly different; and (3) recall bias existed since the participants were asked to recall the remote dietary intake during the 12 months before 3 years. However, there was acceptable reproducibility and validity of the FFQ used at intervals of 3 years [31], and a reliable result of the FFQ questioning the diet in the past 10 years [32]; (4) gastric cancer risk was decreased for obese participants in the present study, which could be because of the commonly observed weight loss in gastric cancer patients; and (5) there was more *H. pylori* infection in the controls than in the cases. This may be because of antibiotic treatments for the cases or reduction of *H. pylori* detection due to the progression of gastric cancer; and (6) although numerous confounding factors were considered in the analyses, there may be some residual confounding effects.

## 5. Conclusions

The risk of gastric cancer was significantly elevated in participants with dietary factors irritating the stomach wall, including high meal frequency, overeating, and preference for spicy or salty foods, respectively, as well as each combination of these factors. Moreover, participants with all these dietary factors were associated with an elevated risk of gastric cancer. Consuming healthy foods has always been suggested, but having dietary factors that do not irritate the stomach wall is also greatly important for gastric health.

## Figures and Tables

**Table 1:** General characteristics according to gastric cancer cases and controls.

	Cases (*n* = 308)		Controls (*n* = 308)		*p*-Values ^a^
Sex (*n* (%))					
Men	207	(67.2)	207	(67.2)	1.000
Women	101	(32.8)	101	(32.8)	
Age (years, mean ± SD)	56.9	±11.9	56.2	±11.9	0.440
Body mass index (kg/m^2^, *n* (%))					
Underweight and normal (<22.9)	155	(50.3)	116	(37.7)	0.007
Overweight (23.0–24.9)	61	(19.8)	69	(22.4)	
Obese (≥25.0)	68	(22.1)	100	(32.5)	
Missing	24	(7.8)	23	(7.5)	
Family history of gastric cancer (*n* (%))					
No	261	(84.7)	278	(90.3)	0.038
Yes	47	(15.3)	30	(9.7)	
Hospital (*n* (%))					
Chungnam National University	124	(40.3)	124	(40.3)	1.000
Hanyang University Guri	184	(59.7)	184	(59.7)	
Education (*n* (%))					
≤Elementary school	90	(29.2)	92	(29.9)	0.913
Middle school	52	(16.9)	49	(15.9)	
High school	101	(32.8)	106	(34.4)	
≥College	36	(11.7)	38	(12.3)	
Missing	29	(9.4)	23	(7.5)	
Smoking (*n* (%))					
Never	109	(35.4)	126	(40.9)	0.338
Past	93	(30.2)	89	(28.9)	
Current	106	(34.4)	93	(30.2)	
Drinking (*n* (%))					
Never	105	(34.1)	103	(33.4)	0.036
Past	54	(17.5)	39	(12.7)	
<20 g/day for women or <40 g/day for men	79	(25.7)	109	(35.4)	
≥20 g/day for women or ≥40 g/day for men	70	(22.7)	57	(18.5)	
*H. pylori* infection (*n* (%))					
Negative	109	(35.4)	67	(21.8)	<0.001
Positive	97	(31.5)	141	(45.8)	
Missing	102	(33.1)	100	(32.5)	
Total energy intake (kcal/day, mean ± SD)	1869.6	±740.5	1698.9	±619.6	0.002
Fruit intake ^b^ (g/day, mean ± SD)	138.6	±177.9	135.8	±204.5	0.858
Vegetable intake ^c^ (g/day, mean ± SD)	63.9	±82.3	51.8	±48.0	0.026

^a^*p*-values by chi-squared test for categorical variables or *t*-test for continuous variables. ^b^ Included fresh fruits, but excluded fruit juice and canned fruits. ^c^ Contained raw and cooked vegetables.

**Table 2:** Odds ratios (ORs) and 95% confidence intervals (95% CIs) of gastric cancer by general risk factors.

	Cases (*n* = 308)	Controls (*n* = 308)	OR (95% CI) ^a^
Body mass index			
Underweight and normal (<22.9)	155	116	1.00 (reference)
Overweight (23.0–24.9)	61	69	0.67 (0.43–1.04)
Obese (≥25.0)	68	100	0.52 (0.35–0.78)
Family history of gastric cancer			
No	261	278	1.00 (reference)
Yes	47	30	1.74 (1.04–2.91)
Education			
≤Elementary school	90	92	1.00 (reference)
Middle school	52	49	1.33 (0.78–2.29)
High school	101	106	1.12 (0.69–1.84)
≥College	36	38	1.31 (0.69–2.48)
Smoking			
Never	109	126	1.00 (reference)
Past	93	89	1.39 (0.78–2.49)
Current	106	93	1.51 (0.87–2.63)
Drinking			
Never	105	103	1.00 (reference)
Past	54	39	1.49 (0.84–2.65)
<20 g/day for women or <40 g/day for men	79	109	0.75 (0.47–1.18)
≥20 g/day for women or ≥40 g/day for men	70	57	1.22 (0.72–2.07)
*H. pylori* infection			
Negative	109	67	1.00 (reference)
Positive	97	141	0.42 (0.27–0.64)

^a^ Mutually adjusted for sex (men or women), age (years, continuous), body mass index (<22.9, 23.0–24.9, ≥25.0 kg/m^2^, or missing), family history of gastric cancer (no or yes), hospital (Chungnam National University Hospital or Hanyang University Guri Hospital), education (≤elementary school, middle school, high school, ≥college, or missing), smoking (never, past, or current), drinking (never, past, <20 g/day for women or <40 g/day for men, or ≥20 g/day for women or ≥40 g/day for men), and *H. pylori* infection (negative, positive, or missing).

**Table 3:** Odds ratios (ORs) and 95% confidence intervals (95% CIs) of gastric cancer by dietary factors irritating the stomach wall.

	Cases (*n* = 308)	Controls (*n* = 308)	OR (95% CI) ^a^
Meal frequency ^b^			
Low frequency (≤3 times/day)	127	170	1.00 (reference)
High frequency (>3 times/day)	181	138	1.96 (1.38–2.79) **
Overeating			
Low amount of rice intake	232	259	1.00 (reference)
High amount of rice intake	76	48	1.96 (1.25–3.07) **
Preference for spicy or salty foods			
Dislike (both)	96	119	1.00 (reference)
Like (either or both)	211	189	1.54 (1.06–2.22) *

^a^ Adjusted for sex (men or women), age (years, continuous), body mass index (<22.9, 23.0–24.9, ≥25.0 kg/m^2^, or missing), family history of gastric cancer (no or yes), hospital (Chungnam National University Hospital or Hanyang University Guri Hospital), education (≤elementary school, middle school, high school, ≥college, or missing), smoking (never, past, or current), drinking (never, past, <20 g/day for women or <40 g/day for men, or ≥20 g/day for women or ≥40 g/day for men), *H. pylori* infection (negative, positive, or missing), fruit intake (g/day, continuous), and vegetable intake (g/day, continuous). ^b^ Frequency of staple food intake consisting of rice and noodles and dumplings. ** *p* < 0.01, * *p* < 0.05.

**Table 4:** Odds ratios (ORs) and 95% confidence intervals (95% CIs) of gastric cancer by combinations of dietary factors irritating the stomach wall.

	Cases (*n* = 308)	Controls (*n* = 308)	OR (95% CI) ^a^
Meal frequency ^b^/Overeating ^c^			
Low frequency/Low amount	100	137	1.00 (reference)
Low frequency/High amount	27	32	1.42 (0.76–2.66)
High frequency/Low amount	132	122	1.74 (1.17–2.58) **
High frequency/High amount	49	16	5.40 (2.71–10.75) **
Meal frequency ^b^/Preference for spicy or salty foods ^d^			
Low frequency/Dislike	44	65	1.00 (reference)
Low frequency/Like	83	105	1.21 (0.72–2.03)
High frequency/Dislike	52	54	1.44 (0.80–2.59)
High frequency/Like	128	84	2.73 (1.62–4.61) **
Overeating ^c^/Preference for spicy or salty foods ^d^			
Low amount/Dislike	77	105	1.00 (reference)
Low amount/Like	155	154	1.53 (1.02–2.29) *
High amount/Dislike	19	14	1.99 (0.88–4.51)
High amount/Like	56	34	2.81 (1.56–5.05) **

^a^ Adjusted for sex (men or women), age (years, continuous), body mass index (<22.9, 23.0–24.9, ≥25.0 kg/m^2^, or missing), family history of gastric cancer (no or yes), hospital (Chungnam National University Hospital or Hanyang University Guri Hospital), education (≤elementary school, middle school, high school, ≥college, or missing), smoking (never, past, or current), drinking (never, past, <20 g/day for women or <40 g/day for men, or ≥20 g/day for women or ≥40 g/day for men), *H. pylori* infection (negative, positive, or missing), fruit intake (g/day, continuous) and vegetable intake (g/day, continuous). ^b^ Frequency of staple food intake consisting of rice and noodles and dumplings; low frequency as ≤ 3 times/day and high frequency as >3 times/day. ^c^ Low amount of rice intake as not overeating; high amount of rice intake as overeating. ^d^ Dislike as not preferred both spicy and salty foods; like as preferred spicy foods, salty foods, or both. ** *p* < 0.01, * *p* < 0.05.

**Table 5:** Odds ratios (ORs) and 95% confidence intervals (95% CIs) of gastric cancer by total scores of dietary factors irritating the stomach wall.

	Cases (*n* = 308)	Controls (*n* = 308)	OR (95% CI) ^a^
Total scores of the dietary factors			
0 (healthier)	37	54	1.00 (reference)
1	110	145	1.25 (0.74–2.10)
2	124	95	2.39 (1.38–4.14) **
3 (unhealthier)	36	13	6.35 (2.73–14.79) **

A higher score presenting as unhealthy and lower score as healthy; a total score ranged from 0 to 3 as whole numbers, which is the sum of the three categories; specifically, a score of 0 as low meal frequency (≤3 times/day), not overeating, and not preferred spicy or salty foods, respectively; and a score of 1 as high meal frequency (>3 times/day), overeating, and preferred spicy or salty foods, respectively. ^a^ Adjusted for sex (men or women), age (years, continuous), body mass index (<22.9, 23.0–24.9, ≥25.0 kg/m^2^, or missing), family history of gastric cancer (no or yes), hospital (Chungnam National University Hospital or Hanyang University Guri Hospital), education (≤elementary school, middle school, high school, ≥college, or missing), smoking (never, past, or current), drinking (never, past, <20 g/day for women or <40 g/day for men, or ≥20 g/day for women or ≥40 g/day for men), *H. pylori* infection (negative, positive, or missing), fruit intake (g/day, continuous) and vegetable intake (g/day, continuous). ** *p* < 0.01.

## Data Availability

The data presented in this study are available on request from the corresponding author. The data are not publicly available because of the privacy of the subjects.
